# Supplementary material for: TMEM132C rs7296262 Single-Nucleotide Polymorphism Is Significantly Associated with Nausea Induced by Opioids Administered for Cancer Pain and Postoperative Pain
Source: Int J Mol Sci. 2024 Aug 14;25(16):8845. doi: 10.3390/ijms25168845 (PMC11354332; doi:10.3390/ijms25168845)
Supplement: Supplementary file 1 [file ijms-25-08845-s001.zip › Kang et al supplemental_TMEM132C_final.pdf]

International Journal of *Molecular Sciences*

MDPI

Supplementary Materials

TMEM132C rs7296262 single-nucleotide polymorphism is significantly associated with nausea induced by opioids administered for cancer pain and postoperative pain

Yuna Kang [1,2], Daisuke Nishizawa [1], Seii Ohka [1], Takeshi Terui [3], Kunihiko Ishitani [3], Ryozo Morino [4], Miyuki Yokota [5,6], Junko Hasegawa [1], Kyoko Nakayama [1], Yuko Ebata [1], Kyotaro Koshika [2], Tatsuya Ichinohe [2] and Kazutaka Ikeda [1,7,*]

Table S1. Background characteristics of patients who developed nausea in the HS group and information related to the opioid administration and cancer pain.

	n	**Minimum**	**Maximum**	**Mean**	**SD**	**Median**
Gender						
male	66					
female	72					
Age (year)	138	34	93	69.413	12.615	70
Height (cm)	138	137	176	158.007	8.828	159.000
Weight (kg)	138	30	110	50.019	11.745	49.000
Drinking						
absence	100					
presence	35					
Smoking history						
absence	81					
presence	54					
Morphine (mg)[a]	138	3.75	1134	101.721	161.967	56.000
Morphine (mg/kg)[b]	138	0.08	18.9	1.994	2.862	1.071
Neuropathic pain						
absence	104					
presence	34					

[a]Total dose of analgesics as a daily average equivalent to oral morphine.

[b]Total dose of analgesics as a daily average per body weight equivalent to oral morphine.

Table S2. Background characteristics of patients without nausea in the HS group and information related to the opioid administration and cancer pain.

	n	Minimum	Maximum	Mean	SD	Median
Gender						
male	97					
female	96					
Age (year)	193	20	94	72.509	11.934	74
Height (cm)	193	140	178	157.558	8.193	158.000
Weight (kg)	193	33	90	51.109	10.902	50.000
Drinking						
absence	142					
presence	45					
Smoking history						
absence	100					
presence	87					
Morphine (mg)[a]	193	3.75	1750	71.625	152.273	30.000
Morphine (mg/kg)[b]	193	0.08	32.4	1.464	3.039	0.604
Neuropathic pain						
absence	143					
presence	50					

[a]Total dose of analgesics as a daily average equivalent to oral morphine.

[a]Total dose of analgesics as a daily average equivalent to oral morphine.

[b]Total dose of analgesics as a daily average per body weight equivalent to oral morphine.

Table S3. Background characteristics of patients who developed nausea in the CIH group and information related to the surgery and anesthesia.

	n	Minimum	Maximum	Mean	SD	Median
Gender						
male	206					
female	644					
Age (year)	850	22	89	57.035	13.567	58
Height (cm)	850	134.4	183.9	159.151	7.664	158.600
Weight (kg)	850	29.9	118	55.595	11.311	53.800
Body mass index (kg/m^2)	850	13.87	45.57	21.882	3.794	21.245
Smoking history						
absence	509					
presence	341					
Motion sickness						
absence	399					
presence	450					
Total dosage of fentanyl (μg)	850	0	800	217.275	131.081	200.000
Total dosage of remifentanil (μg)	850	0	17000	2889.765	2391.726	2300.000
Use of pentazocine						
absence	258					
presence	592					
Use of opioid after anesthesia						
absence	357					
presence	493					
Anesthesia method						
TIVA[a]	218					
inhalation anesthetic	632					
Pain						
absence	233					
presence	617					

[a]General anesthesia using total intravenous anesthesia.

Table S4. Background characteristics of patients without nausea in the CIH group and information related to the surgery and anesthesia.

	n	Minimum	Maximum	Mean	SD	Median
Gender						
male	494					
female	677					
Age (year)	1171	20	93	57.594	13.836	59
Height (cm)	1171	126.5	188.5	161.339	8.378	160.900
Weight (kg)	1171	32.2	109.1	58.011	11.124	56.800
Body mass index (kg/m^2)	1171	14.46	37.92	22.207	3.447	21.790
Smoking history						
absence	550					
presence	621					
Motion sickness						
absence	723					
presence	448					
Total dosage of fentanyl (μg)	1171	0	1000	184.952	127.410	200.000
Total dosage of remifentanil (μg)	1171	0	24300	2472.502	2173.196	2000.000
Use of pentazocine						
absence	580					
presence	591					
Use of opioid after anesthesia						
absence	699					
presence	472					
Anesthesia method						
TIVA[a]	427					
inhalation anesthetic	744					
Pain						
absence	381					
presence	790					

[a]General anesthesia using total intravenous anesthesia.

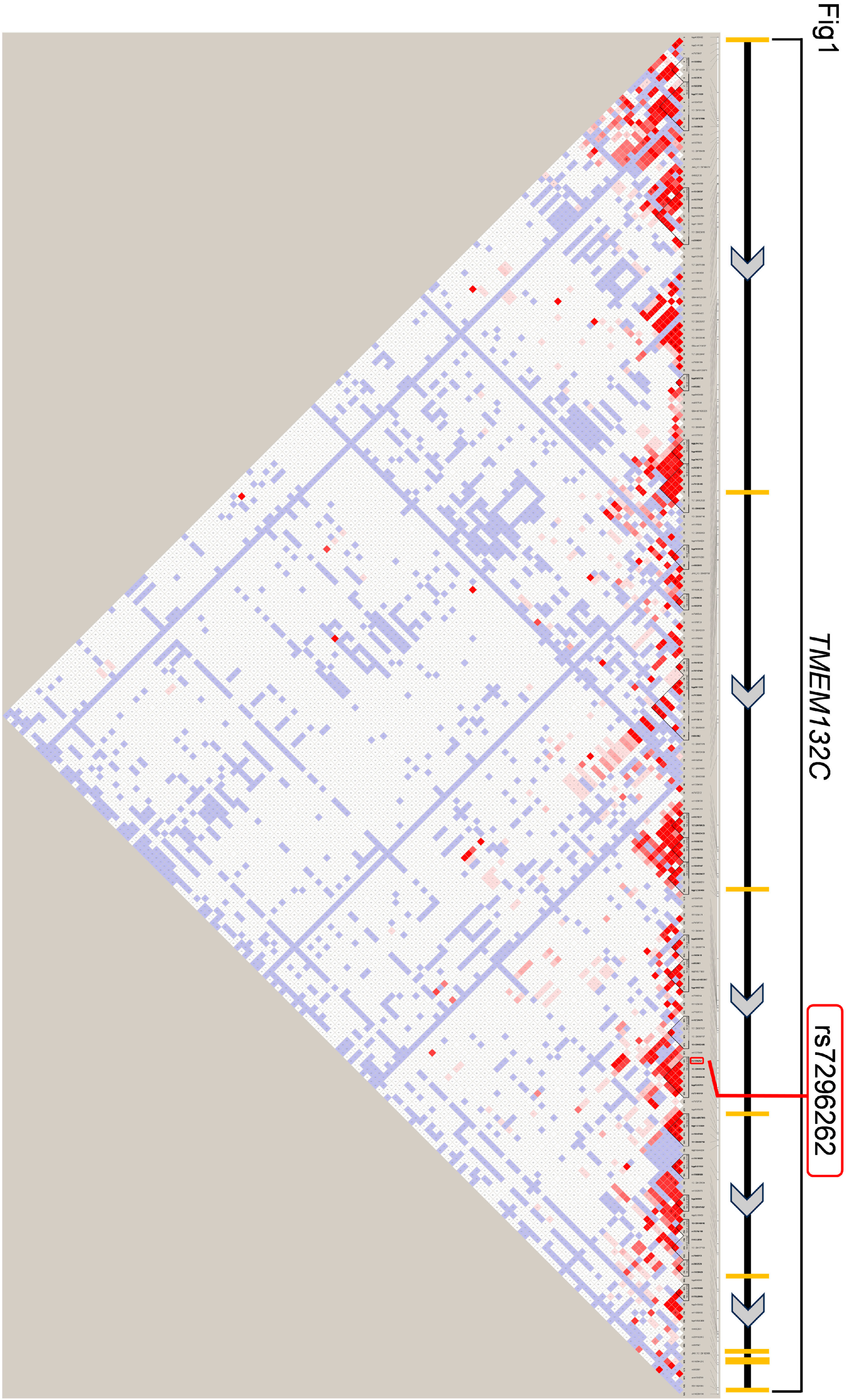

Figure S1. State of LD among SNPs in the *TMEM132C* gene region (LD plot-r^2) in the HS group. White boxes represent $D' < 1$, log of likelihood odds ratio (LOD) < 2. Pink and red boxes represent $D' < 1$ and LOD ≥ 2. Blue boxes represent $D' = 1$ and LOD < 2. Bright red boxes represent $D' = 1$ and LOD ≥ 2. The solid horizontal line above the LD plot represents the *TMEM132C* gene. The yellow boxes in the structure of the *TMEM132C* gene represent exons, and the solid lines represent untranslated regions or introns. The gray arrows represent the direction of transcription. The red squares represent the SNP of interest in this study.

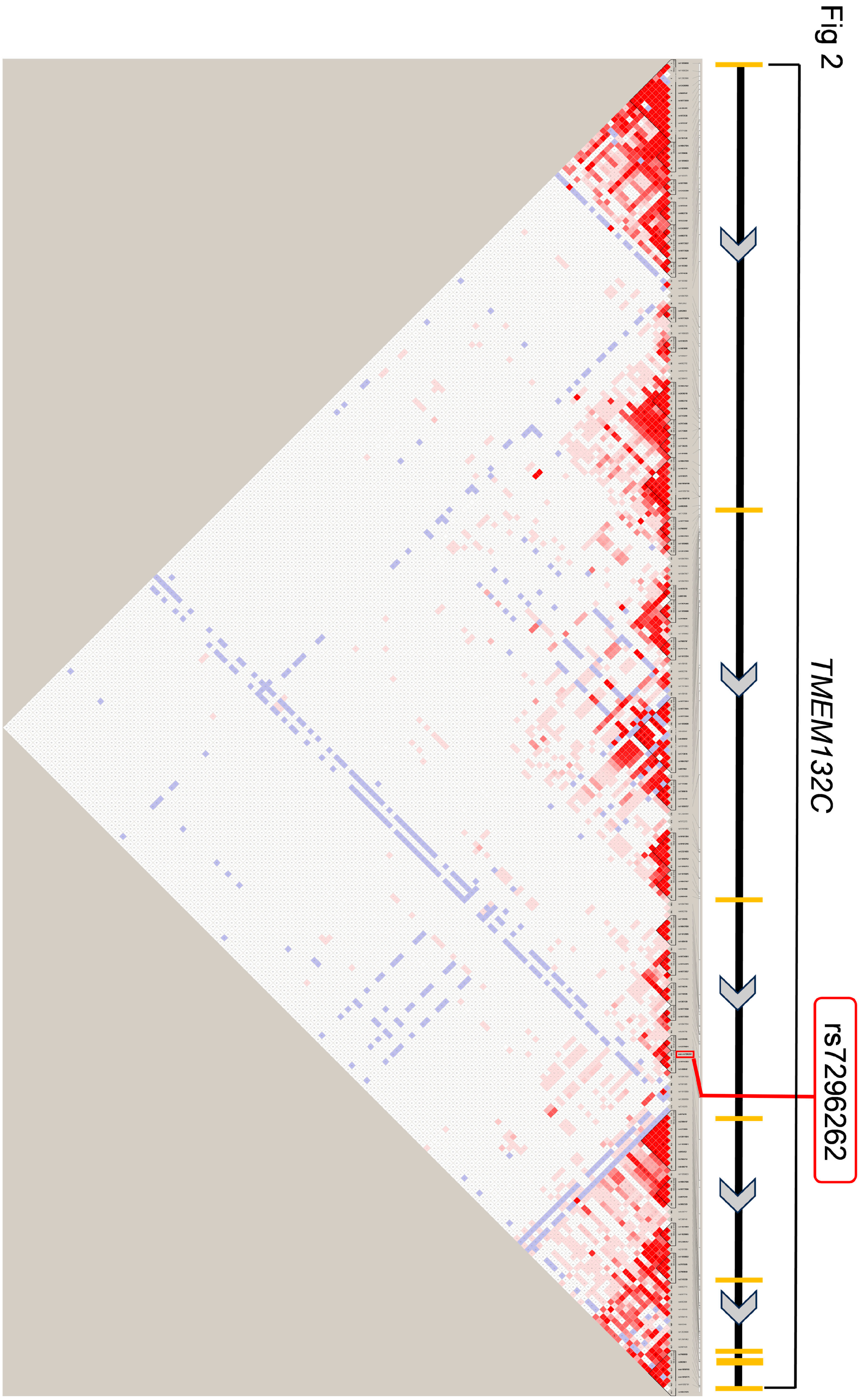

Figure S2. State of LD among SNPs in the *TMEM132C* gene region (LD plot-r^2) in the CIH group. A detailed explanation is given in the Figure S1 legend.

Table S5. Human permissive enhancers around rs7296262 SNP.

Location	Score for human permissive enhancers
chr12:129054843-129055190	2
chr12:129089156-129089231	6

Data were extracted from 100-kbp upstream and downstream of the rs7296262 (chr12:129095072) SNP on the plus strand.

Table S6. Cap-analysis gene expression (CAGE) signal around the rs7296262 SNP.

Location	Expression score
chr12:129043329-129043456	2.16
chr12:129043457-129043584	6.16
chr12:129043841-129043968	0.53
chr12:129055105-129055232	0.77
chr12:129089153-129089280	0.55
chr12:129149313-129149440	1.8
chr12:129175553-129175680	0.63
chr12:129186945-129187072	4.07
chr12:129187073-129187200	2.62
chr12:129187201-129187328	7.44

Data were extracted from 100-kbp upstream and downstream of the rs7296262 (chr12:129095072) SNP on the plus strand.

Table S7. Protein-coding transcripts that start around the rs7296262 SNP.

Location	Transctipt	Protein
chr12:129028499-129192454	ENST00000315208.8	ENSP00000324458
chr12:129178450-129192456	ENST00000537538.1	ENSP00000438477

Data were extracted from 100-kbp upstream and downstream of the rs7296262 (chr12:129095072) SNP on the plus strand.
